# Circulating Cell-Free DNA Is Associated With Cognitive Outcomes

**DOI:** 10.1093/geroni/igaa057.1671

**Published:** 2020-12-16

**Authors:** Danielle Feger, Lolita Nidadavolu, Esther Oh, Peter Abadir, Alden Gross

**Affiliations:** 1 Johns Hopkins University, Baltimore, Maryland, United States; 2 Johns Hopkins University School of Medicine, Baltimore, Maryland, United States; 3 Johns Hopkins Bloomberg School of Public Health, Baltimore, Maryland, United States

## Abstract

Cell death is a mechanism by which aging tissues are able to maintain homeostasis. DNA of nuclear and mitochondrial origin is released into circulation following apoptosis or necroptosis and can be quantified in the blood as circulating cell-free DNA (ccf-DNA). We hypothesized that higher levels of ccf-DNA would be associated with worse cognitive function. Ultra-sensitive digital PCR was used to measure ccf-DNA in participants from the Rush Alzheimer’s Disease Center Religious Orders Study/Memory and Aging Project. Global cognitive function was derived from a composite of 19 tests on a neuropsychiatric battery. A total of 885 ccf-DNA samples were analyzed from N=624 participants. Generalized estimating equations were used to estimate the cross-sectional association between ccf-DNA and global cognition scores, while latent growth models were used to estimate the longitudinal association between ccf-DNA and global cognition scores. Multinomial logistic regression was used to estimate the odds of having mild cognitive impairment (MCI) or dementia at last study visit relative to normal cognition, based on levels of ccf-DNA. Higher ccf-DNA levels were associated with lower global cognition score (-0.10, [-0.18, -0.02]) cross-sectionally. Each 1-standard deviation increase in ccf-DNA was associated with more rapidly declining global cognitive function over time (-0.11, [-0.19, -0.03]). A dose-response relationship was observed between increasing levels of ccf-DNA and odds of MCI (odds ratio [OR] = 1.08, [0.83, 1.41]) and dementia (OR = 1.29, [1.06, 1.57]). Our results suggest that ccf-DNA may serve as a biomarker of global cognitive decline and dementia risk.

